# Dietary patterns and hepatocellular carcinoma risk: a systematic review and meta-analysis of cohort and case–control studies

**DOI:** 10.1186/s12986-024-00822-y

**Published:** 2024-07-11

**Authors:** Wenxi Shu, Ling Liu, Jiaojiao Jiang, Qinghua Yao

**Affiliations:** 1 Second Clinical Medical College, Zhejiang Chinese Medical University, Hangzhou, 310053 China; 2 The Second Affiliated Hospital of Zhejiang, Chinese Medical University, Xinhua Hospital of Zhejiang Province, Hangzhou, 310005 Zhejiang China; 3 Hangzhou Institute of Medicine (HIM), Chinese Academy of Sciences, Hangzhou, 310022 Zhejiang China; 4 Key Research Laboratory of the Pathological Mechanism of Intestinal Disease ‘Inflammation-Cancer’ Transformation, Zhejiang, 310005 China

**Keywords:** Diet pattern, Hepatocellular carcinoma, HCC, Systematic review, Meta-analysis

## Abstract

**Background:**

Globally, HCC presents a significant health burden, characterized by high incidence and mortality rates. Epidemiological studies have increasingly suggested a link between dietary patterns and the risk of hepatocellular carcinoma (HCC), yet consensus on this relationship remains elusive.

**Objective:**

This study aims to synthesize existing literature and provide a comprehensive analysis of the association between dietary patterns and HCC risk through meta-analytical methods.

**Methods:**

A systematic search of PubMed, Embase, and the Cochrane Library databases was conducted to identify studies examining common dietary patterns in relation to HCC, published up to August 2023. Study quality was rigorously evaluated using the Newcastle–Ottawa Scale. We employed a random effects model to synthesize effect sizes, calculating hazard ratios (HRs) and 95% confidence intervals (CIs).

**Results:**

We identified 13 papers, of these 10 investigating a priori dietary patterns(index-based dietary patterns) and 3 focusing on a posterior dietary patterns (data-driven dietary patterns). Analysis of a priori dietary patterns revealed that higher scores in the Healthy Eating Index (HEI) & alternative HEI (HR = 0.67, 95% CI: 0.54–0.85), Dietary Approaches to Stop Hypertension (DASH) (HR = 0.77, 95% CI: 0.66–0.91), and the Mediterranean diet (HR = 0.65, 95% CI: 0.56–0.75) were associated with a reduced risk of HCC. Conversely, pro-inflammatory dietary patterns were linked with an increased risk (HR = 2.21, 95% CI: 1.58–3.09). In a posterior dietary patterns, a vegetable-based diet was negatively correlated with HCC risk (HR = 0.63, 95% CI: 0.49–0.81).

**Conclusion:**

This meta-analysis underscores a significant association between dietary patterns and the risk of HCC. Adherence to healthy dietary patterns characterized by high in vegetables, whole grains, legumes, nuts, and low in red and processed meats may confer a protective effect against HCC, whereas inflammatory diets appear to elevate risk.

**Supplementary Information:**

The online version contains supplementary material available at 10.1186/s12986-024-00822-y.

## Introduction

Globally, hepatocellular carcinoma (HCC) ranks prominently as a leading cause of cancer-related mortality and morbidity [1]. Predominantly prevalent in East Asia, Southeast Asia, and certain regions of North and West Africa, with notable prevalence in China, HCC presents a significant global health concern [2]. The key risk factors of HCC encompass chronic Hepatitis B Virus (HBV) and Hepatitis C Virus (HCV) infections, exposure to aflatoxin-contaminated foodstuffs, excessive alcohol consumption, obesity, type 2 diabetes, and smoking. These factors are known to precipitate Chronic Liver Disease (CLD), a precursor to HCC. However, a considerable proportion of HCC cases arise in individuals without these established risk factors, indicating the potential involvement of additional etiological elements [3]. Recent epidemiological studies have underscored the potential role of dietary influences in the pathogenesis of HCC. Observational data suggest a possible inverse relationship between HCC risk and increased consumption of vegetables [4, 5], fruits [6, 7], white meat or fish [8, 9], and dairy products [7], alongside a reduced intake of red meat and saturated fats [10, 11].

The complexity of dietary habits, characterized by the interrelated consumption of various food items, presents challenges in isolating the impact of individual dietary components [12, 13]. Dietary pattern analysis, encompassing both 'a posterior' (data-driven) and 'a priori' (index-based) methodologies, has emerged as a pivotal approach in nutritional epidemiology. The posterior approach, driven by population-specific data, employs statistical techniques such as factor analysis and principal component analysis. In contrast, the a priori method relies on predefined criteria, potentially rooted in dietary guidelines, cultural practices, and biomarkers, to assess adherence to specific dietary patterns [14]. While dietary pattern analysis is increasingly recognized as an effective tool for evaluating the aggregate impact of diet on health, the specific connection between dietary patterns and HCC risk is still an active area of research with indeterminate outcomes. Several observational studies variably report the protective effects of certain diets [15–17], such as the Mediterranean diet, against HCC, with others noting no significant associations [18, 19]. One systematic review, published in 2021, suggested a potential role of diet in the development of HCC. However, it only provided a qualitative description and did not conduct further meta-analysis [20]. A recent meta-analysis has focused on associations between dietary patterns and several cancer risks, but did not include liver cancer [21].

Responding to the emergence of new research and the need for a more precise estimate in this area, we have executed an extensive systematic review and meta-analysis, encompassing cohort and case–control studies. This initiative is designed to amalgamate and strengthen the prevailing evidence on the relationship between dietary patterns and hepatocellular carcinoma (HCC) risk, thereby seeking to clarify and distill the accumulated knowledge in this sphere.

## Method

The meta-analysis strictly followed PRISMA (Preferred Reporting Items for Systematic Reviews and Meta-Analyses) guidelines, with the manuscript structured accordingly. This study protocol was proactively registered with PROSPERO (International Prospective Register of Systematic Reviews) under the registration number CRD42022349181. The research question was developed according to the PICOS (Population, Intervention, Comparison, Outcomes, and Study Design) criteria (Table 1).

**Table 1:** The PICOS criteria employed for the inclusion and exclusion of studies

Parameter	Inclusion Criteria
Population	Adults above the age of 18 years and free of liver cancer at baseline for cohort study
Intervention/exposures	Highest category of dietary pattern score
Comparison	Lowest category of dietary pattern score
Outcomes	Incidence of liver cancer
Study design	Case–control studies and cohort studies

### Search strategy

A comprehensive search was systematically performed in databases including PubMed, EMBASE, and the Cochrane Library, targeting English-language publications prior to August 2023. To ascertain comprehensive retrieval of pertinent studies, a strategic combination of keywords and phrases was employed: (("liver" OR "hepatic" OR "hepatocellular" OR "hepatoma") AND ("neoplasm" OR "cancer" OR "carcinoma" OR "tumor") AND ("dietary pattern" OR "eating pattern" OR "food pattern" OR "diet pattern" OR "diet" OR "dietary")). In addition to database searches, an in-depth analysis of bibliographies from the selected articles and relevant review papers was conducted to capture any additional significant studies.

### Inclusion criteria

Inclusion criteria for this study were as follows: (1) The study design was either a case–control or cohort study; (2) The exposure of interest was various dietary patterns or scores; (3) Measurement of HCC incidence as the outcome, with all HCC cases confirmed via histopathological biopsy or other standard methods, and controls comprising HCC-free adults, inclusive of all HCC types like hepatocellular carcinoma and intrahepatic cholangiocarcinoma; (4) Provision of risk estimates—relative risks (RRs), hazard ratios (HRs), or odds ratios (ORs)—for the most versus least adherent dietary pattern groups, along with the corresponding 95% confidence intervals (CIs).

### Study selection process

**Fig. 1:**
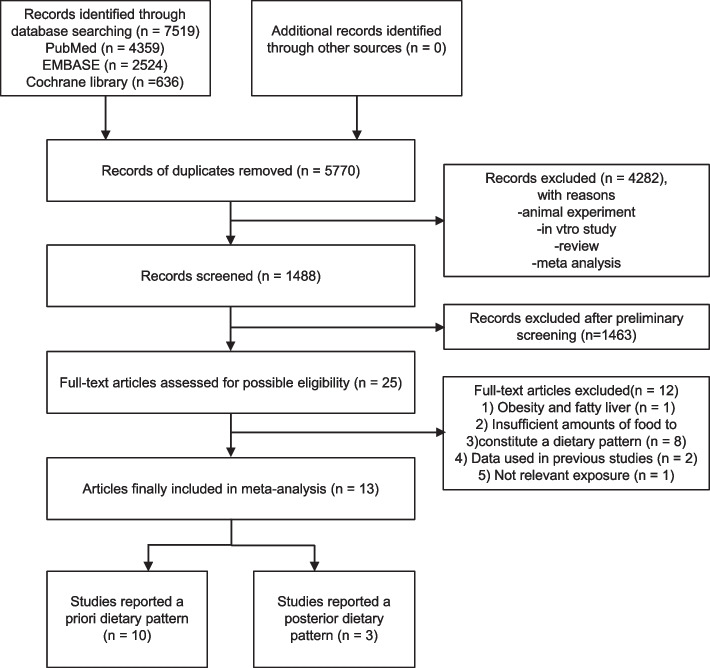
Flowchart of the systematic review process

Two reviewers independently screened titles and abstracts of retrieved studies, excluding those not meeting set inclusion criteria. Unclear cases were resolved through full-text review and discussion. The study selection process is illustrated in Fig. 1.

### Data extraction and quality assessment

The characteristics extracted for analysis, as delineated in Table 2, encompassed a range of parameters including the surname of the first author, year of publication, geographical location of the study, the study's methodological design, the duration of follow-up or data collection period, total sample size, demographic composition by gender, age range at baseline, methods employed for deducing dietary patterns, the dietary patterns themselves, and any potential confounding variables addressed in multivariate analyses. Detailed descriptions of the dietary assessment tools employed for evaluating both a posterior and a priori dietary patterns are presented in Tables 3 and 4. These tables include the terminology of the dietary patterns, a breakdown of the specific food items or nutrients comprising each a posterior dietary pattern, and the scoring methodologies utilized for a priori dietary patterns. Additionally, risk estimates such as Odds Ratios (ORs), Hazard Ratios (HRs), and Relative Risks (RRs), along with 95% Confidence Intervals for the extreme categories of dietary pattern scores in the most comprehensively adjusted models, and the *p*-values for observed trends (as applicable) were also reported.

**Table 2:** Overview of thirteen epidemiological studies investigating the association between dietary patterns and liver cancer

Author	Year	Location	Study design (cohort name)	data collection period	Sample size	sex	Age of participants^a^ (year)	Body mass index^b^ (kg/m^2^)	Type of dietary pattern	Variables for adjustment
Bogumil	2019	US	Cohort (Multiethnic centre /MEC)	1993–2013	169,806 (605 cases)	78,450 M 91,356 F	45–75	NA	A priori: HEI2010, AHEI2010, aMED, DASH	Age, sex, race/ethnicity, BMI, diabetes, smoking status, and total energy
Chen	2018	China	Case–control (Sun Yat-sen University Cancer Center)	Sep 2013- Oct 2017	720 cases/ 720 controls	613 M 107 F	Case: 58.2 ± 8.8 Control: 58.4 ± 8.1	Case: 22.8 ± 3.2 Control: 23.7 ± 3.1	A priori: CHEI, HEI-2015	Age, sex, BMI, physical activity, education level, household monthly income per capita, smoker status, alcohol consumption, history of diabetes, HBV seropositivity, and non-alcohol energy intake
Lan	2018	China	Case–control (Sun Yat-sen University Cancer Center)	Sep 2013- Aug 2016	782 cases/ 782 controls	680 M 102 F	Case: 52.71 ± 11.27 Control: 53.02 ± 10.20	Case: 22.81 ± 3.31 Control: 23.25 ± 3.23	A posterior: UPDP, TCDP, MPFP	Sex, age, BMI, marital status, education level, income level, smoking, alcohol use, tea drinking, physical activity, multivitamin use, and hypertension and diabetes status
Li	2014	US	Cohort (NIH-AARP Diet and Health Study)	1995–2006	494,942 (509 cases)	295,283 M 199,659 F	50–71	NA	A priori: HEI2010, aMED	Age, sex, alcohol intake, smoking, body mass index, education, race, diabetes, usual activity throughout the day, vigorous physical activity, and total energy intake
Luu	2020	Singapore	Cohort (Singapore Chinese Health Study/SCHS)	17.6 (5.3) years	63,257 (561 cases)	27,293 M 34,028 F	45–74	Case: 24.0 ± 3.6 Non-case: 23.1 ± 3.3	A priori: AHEI2010, aMED, DASH, HDI	Age, gender, dialect group, level of education, year of enrollment, BMI, smoking status, alcohol drinking, diabetes and total energy intake
Ma	2019	US	Cohort (Nurses’ Health Study/ NHS and Health Professionals Follow-up Study/HPFS)	32 years	173,229 (160 cases)	M (HPFS): 51,529 F (NHS): 121,700	M: 40–75 F: 30–55	NA	A priori: AHEI-2010, AMED, DASH	Race, physical activity level, BMI, smoking, regular aspirin use, total calorie intake, type 2 diabetes, alcohol intake(DASH)
Moussa	2021	US	Case–control (The University of Texas MD Anderson Cancer Center)	Mar 2001- Mar 2018	641 cases/ 1002 controls	The M: F ratio was 2.8:1 among cases	Cases: 62.9 ± 10.9 Controls: 60.0 ± 10.7	NA	A posterior: Vegetable-based, Western	Sex, age, race, education, alcohol consumption, cigarette smoking, diabetes, BMI, family history of cancer, and HBV/HCV infection
Shivappa	2016	Italy	Case–control (Italian multi-centre)	Jan 1999- July 2002	185 cases/ 404 controls	NA	Case: 43–84 Control: 40–82	NA	A priori: DII	Age, sex, study centre, education, BMI, smoking, physical activity, serum markers of hepatitis B and C infection and energy intake
Turati	2014	Italy and Greece	Case–control (Italy, Greece)	Italy: 1999–2002 Greece: 1995–1998	518 cases/ 772 controls	Cases: M: 432, F: 86 Control: M: 579, F: 193	NA	NA	A priori: MDS	Center, age, sex, education, BMI, smoking, history of diabetes, non-alcohol energy intake, and HBsAg and/or anti-HCV positivity
Wang	2018	South China	Case–control (Sun Yat-sen University Cancer Center)	Sep 2013- Oct 2017	659 cases/ 659 controls	Cases: M: 568, F: 91 Control: M: 568, F: 91	Cases vs. controls: 45.7% vs. 44.6% for ≥ 60 years	Cases vs. controls: 69.2% vs. 54.9% for BMI < 24 kg/m^2^	A priori: DII	Age, energy intake, body mass index, physical activity, marital status, education, household income, smoking status, and hepatitis B virus (HBV) infection status
Yang	2021	US	Cohort (the Nurses’ Health Study/ NHS and the Health Professionals Follow-up Study/HPFS)	Average follow-up of 25.6 years	119,316 (142 cases)	M: 49,261 F: 70,055	NA	NA	A priori: EDIP, EDIH, EDIR	Age, sex, race, physical activity, smoking status, aspirin use, and total energy intake
Zhang	2013	China	Cohort (Shanghai Women’s Health Study/SWHS and the Shanghai Men’s Health Study/SMHS)	During an average follow-up of 10.9 (SWHS) or 5.5 (SMHS) years	132,837 (267 cases)	M: 60,207 F: 72,966	M: 56.1 ± 10.3 F: 52.6 ± 9.1	SMHS: Case: 23.3 ± 0.3 Non-case: 23.7 ± 0.0 SWHS: Case: 24.7 ± 0.3 Non-case: 24.0 ± 0.0	Data-driven: Vegetable-based; Fruit-Base; Meat-based	Age at enrolment, BMI, total energy intake, sex, family income level, education level, family history of liver cancer in first-degree relatives, history of chronic viral hepatitis, chronic liver disease or cirrhosis, diabetes, and cholelithiasis or cholecystectomy, and Vitamin C, vitamin E, or multivitamin supplement use
Zhong	2020	US	Cohort (PLCO Cancer Screening Trial)	13 years	103,902 (120 cases)	M: 51,420 F: 52,482	62.5 ± 5.5	27.3 ± 4.8	A Priori: DII	Age, sex, educational level, BMI, energy intake from diet, smoking status, alcohol drinking status, diabetes, and family history of liver cancer

*Abbreviation:* HEI Healthy Eating Index, *aHEI* Alternative Healthy Eating Index, *aMED* Alternate Mediterranean Diet, *DASH* Dietary Approaches to Stop Hypertension, *CHEI* Chinese Healthy Eating Index, *UPDP* urban prudent dietary pattern, *TCDP* traditional Cantonese dietary pattern, *MPFP* high meat and preserved food pattern, *HDI* Heathy Diet Indicator, *DII* dietary inflammatory index, *MDS* Mediterranean diet score, *EDIP* empirical dietary inflammatory pattern, *EDIH* empirical dietary index for hyperinsulinemia, *EDIR* empirical dietary index for insulin resistance, *BMI* body mass index, *NHS* Nurses’ Health Study, *HPFS* Health Professionals Follow-up Study, *SWHS* Shanghai Women’s Health Study, *SMHS* Shanghai Men’s Health Study, *PLCO* Prostate, Lung, Colorectal and Ovarian Cancer Screening Trial

^**a**^Values are mean ± SD or age range

^**b**^Values are mean ± SD/SE

**Table 3:** Characteristics and associations of a posterior dietary patterns with HCC risk

Author	Year	Location	Study design	Dietary assessment instrument	Period of dietary assessment	Dietary pattern and component foods	Main results
Lan	2018	China	Case–control	FFQ (79-item food)	1 year prior to cancer diagnosis	**Urban prudent dietary** **pattern(UPDP):** characterized by high in dairy products, eggs, mushrooms, nuts and soy foods, but low in refined grains **traditional Cantonese dietary pattern (TCDP):** consisting of a high intake of fruit and vegetables, fish, Cantonese soup, and Chinese herb tea **high meat and preserved food pattern (MPFP)**	UPDP: OR = 0.25, 95% CI: 0.18–0.35, *p* < 0.001 TCDP: OR = 0.61, 95% CI: 0.46–0.82, *p* = 0.002 MPFP: OR = 1.98, 95% CI:1.46–2.60, *p* < 0.001
Moussa	2021	US	Case–control	FFQ	1 year prior to cancer diagnosis for cases and prior to recruitment for controls	**Vegetable-based:** dietary pattern characterized by high intake of many vegetables **Western diet:** pattern characterized by high factor loading of red meat, processed meat, snacks, and sweets	Vegetable-based: OR = 0.66, 95% CI: 0.46–0.94, *p* = 0.018 Western diet: OR = 1.79, 95% CI: 1.19–2.69, *p* = 0.012
Zhang	2013	China	Cohort	SWHS: FFQ (77 food items); SMHS: FFQ (81 food items)	1 year previous to FFQ	**Vegetable-based:** characterized by high intake of vegetables; **fruit-based:** characterized by high intake of fresh fruits; **meat-based:** characterized by high intake of meat, poultry, and animal parts (heart, brain, tongue, intestine, etc.)	Vegetable-based: HR = 0.58, 95% CI: 0.40–0.84, *p* = 0.01 Fruit-based: HR = 1.13, 95% CI: 0.78–1.64, *p* = 0.39 Meat-based: HR = 1.18, 95% CI: 0.83–1.69, *p* = 0.51

*Abbreviation* *:* *FFQ* food frequency questionnaire

**Table 4:** Characteristics of a priori dietary patterns and their associations with HCC risk

Author	Year	Location	Study design	Dietary assessment instrument	Period of dietary assessment	Dietary pattern	Main results^a^
Bogumil	2019	US	Cohort	FFQ (> 180 food items)	NA	**HEI-2010** **aHEI-2010** **aMED** **DASH**	HEI-2010: HR = 0.84, 95% CI: 0.64–1.12, *p* = 0.188 aHEI-2010: HR = 0.87, 95% CI: 0.66–1.14, *p* = 0.231 aMED: HR = 0.68, 95% CI: 0.51–0.90, *p* = 0.016 DASH: HR = 0.89, 95% CI: 0.68–1.16, *p* = 0.286
Chen	2018	China	Case–control	FFQ (79-item)	1 year prior to cancer diagnosis for cases and prior to interview for controls	**CHEI** **HEI-2015**	CHEI: OR = 0.43, 95% CI: 0.38–0.50 HEI-2015: OR = 0.47, 95% CI: 0.40–0.55
Li	2014	US	Cohort	FFQ (124-item)	1 year previous to FFQ	**HEI-2010** **aMED**	HEI-2010: HR = 0.72, 95% CI: 0.53–0.97, *p* = 0.03 aMED: HR = 0.62, 95% CI: 0.47–0.84, *p* = 0.0002
Luu	2020	Singapore	Cohort	FFQ (165-item)	NA	**AHEI-2010** **aMED** **DASH** **HDI**	AHEI-2010: HR = 0.69, 95% CI: 0.53–0.89, *p* = 0.02 aMED: HR = 0.70, 95% CI: 0.52–0.95, *p* = 0.06 DASH: HR = 0.67, 95% CI: 0.51–0.87, *p* = 0.004 HDI: HR = 0.85, 95% CI: 0.55–1.09, *p* = 0.04
Ma	2019	US	Cohort	FFQ	NA	**AHEI-2010** **aMED** **DASH**	AHEI-2010: HR = 0.61, 95% CI: 0.39–0.95, *p* = 0.03 AMED: HR = 0.75; 95% CI: 0.49–1.15, *p* = 0.18 DASH: HR = 0.90; 95% CI: 0.59–1.36, *p* = 0.61
Shivappa	2016	Italy	Case–control	FFQ (63-item)	2 years before the date of interview	**DII**	OR = 2.43, 95% CI: 1.27–4.68, *p* = 0.03
Turati	2014	Italy and Greece	Case–control	FFQ	Italian study: 2 years before cancer diagnosis or hospital admission (for controls) Greek study: over a period of 1 year preceding the recognition of symptoms or signs of the present disease	The Mediterranean diet score (MDS)	OR = 0.51, 95% CI: 0.34–0.75, *p* < 0.001
Wang	2018	South China	Case–control	FFQ (79-item)	1 year prior to cancer diagnosis for cases and prior to interview for controls	**DII**	OR = 3.22, 95% CI: 1.30–7.98, *p* = 0.009
Yang	2021	US	Cohort	FFQ	NA	**EDIP** **EDIH** **EDIR**	EDIP: HR = 2.03, 95% CI: 1.31–3.16, *p* = 0.001 EDIH: HR = 1.61, 95% CI: 1.06–2.43, *p* = 0.02 EDIR: HR = 1.62, 95% CI: 1.08–2.42, *p* = 0.02
Zhong	2020	US	Cohort	FFQ (137-item)	1 year previous to FFQ	**DII**	HR = 2.05, 95% CI: 1.23–3.41

^**a**^A comparing highest to lowest adherence groups in the fully adjusted model

The study quality assessment was systematically performed by the Newcastle–Ottawa Scale (NOS). Two independent reviewers appraised each study against three broad criteria: (1) the appropriateness of the study population selection, (2) the comparability of the study groups, and (3) the accuracy of exposure ascertainment in cohort studies or outcome ascertainment in case–control studies. Any differences in assessment were reconciled through discussion to achieve a consensus. Studies that achieved a score of 7 or higher out of a possible 9 points on the NOS were classified as high quality.

### Statistical analysis

In most studies, Hazard Ratios (HRs) were employed to assess the link between dietary patterns and HCC risk, with Relative Risks (RRs), Odds Ratios (ORs), or Incidence Rate Ratios (IRRs) also serving as HR estimates due to low incidence rates [22]. A random effects meta-analysis, chosen for its conservative approach amidst expected heterogeneity [23], was used to calculate overall HRs and their 95% confidence intervals. Heterogeneity was assessed using the Q statistic (significance at *p* < 0.10) and the I^2^ statistic, with an I^2^ over 50% indicating moderate heterogeneity [24]. Publication bias was assessed through funnel plots using Egger’s test.

Subgroup analyses were performed to discern the potential impact of varying scoring criteria within the same dietary pattern. Moreover, sensitivity analyses were performed by systematically excluding individual studies or groups of studies to assess their singular impact on the overall findings. All statistical analyses were executed using STATA version 15.0 (STATA Corp LP, College Station, Texas), with statistical significance set at a *p*-value below 0.05.

## Result

### Literature search and study characteristics

Our preliminary search yielded 7,519 potentially relevant articles. We were left with 1,488 articles following the exclusion of duplicates, reviews, systematic reviews, meta-analyses, and animal studies. Subsequent scrutiny of titles and abstracts led to the further exclusion of 1,463 articles. Of the remaining 25 articles evaluated in full text, 12 were excluded for the following reasons: one did not report HCC risk but instead focused on the relationship between dietary patterns and obesity and fatty liver [25]; eight lacked sufficient food item quantity to constitute a dietary pattern [6, 26–32]; two used data previously employed by the same authors in other studies [33, 34]; and one focused on glycemic load or index as the exposure [35] (Fig. 1). Ultimately, 13 articles were included in the analysis: 10 focusing on a priori dietary patterns and three reporting a posterior dietary patterns.

The characteristics and detailed information of the 13 articles included in this study are presented in Table 2. Of these, six reported on case–control study results, and seven presented findings from cohort studies. Notably, three of these articles each covered two separate cohort studies, while another reported on two case–control studies but only provided the combined ORs of these studies. As a result, this meta-analysis encompassed seven case–control studies and ten cohort studies. These studies were published between 2013 and 2021, conducted in various locations including the United States (*n* = 8), China (*n* = 5), Italy (*n* = 2), Singapore (*n* = 1), and Greece (*n* = 1). The sample sizes of these studies varied widely, ranging from 589 to 494,942, with the number of HCC cases ranging from 118 to 782. In their analyses, these studies adjusted for a range of potential confounders, including age, gender, body mass index, smoking habits, total energy intake, and physical activity.

In this study, to elucidate the types of dietary patterns, four studies utilized a posterior dietary patterns (Table 3), while thirteen studies were based on a priori dietary patterns (Table 4). Among these investigations, six focused on examining the relationship between the Healthy Eating Index (HEI) and the Alternate Healthy Eating Index (AHEI) with the risk of HCC. Four studies concentrated on the Dietary Approaches to Stop Hypertension (DASH) dietary pattern and HCC risk, six evaluated the Mediterranean Diet (MD) and its association with HCC, five analyzed pro-inflammatory diets and HCC risk, and two explored vegetable-based diets in relation to HCC risk, with each of the other dietary patterns being the subject of a single study. Consequently, we conducted a meta-analysis on those dietary patterns that were the focus of more than one study in relation to HCC risk. The Newcastle–Ottawa Scale (NOS) scores for each study are presented in Table 5., with scores ranging from 6 to 9, including 14 studies deemed high-quality and 3 of lower quality. Notably, we observed that most case–control studies did not report non-response rates and did not describe whether blinding was used in the assessment and investigation of exposures.

**Table 5:** Assessment outcomes of case-control and cohort studies in meta-analyses using the Newcastle-Ottawa Scale (NOS)

	**Selection**				**Comparability**	**Exposure**			
Case-control studies	Case definition	Representa-tiveness of the cases	Selection of Controls	Definition of Controls	Control for most important factor and Control for any additional factor	Ascertainment of exposure	Same method of ascertainment for cases and controls	Non-Response rate	Total scores
Chen 2018 [16]	1	1	1	1	2	0	1	0	7
Lan 2018 [36]	1	1	1	1	2	0	1	0	7
Moussa 2021 [17]	1	1	1	1	2	0	1	0	7
Shivappa 2016 [37]	1	1	0	1	2	0	1	0	6
Turati (Italy) 2014	1	1	0	1	2	0	1	0	6
Turati (Greece) 2014	1	1	0	1	2	0	1	0	6
Wang 2018	1	1	1	1	2	0	1	0	7
	**Selection**				**Comparability**	**outcome**			
Cohort studies	Representativeness of the exposed cohort	Selection of the non-exposed cohort	Ascertainment of exposure	Outcome was not present as baseline	Control for most important factor and Control for any additional factor	Assessment of outcome	Adequate follow-up period for outcome	Adequacy of follow up of cohorts	Total scores
Bogumil 2019 [15]	1	1	1	1	2	1	1	0	8
Li 2014 [38]	1	1	1	1	2	1	1	0	8
Luu 2020	1	1	1	0	2	1	1	1	8
Ma (NHS) 2019 [39]	0	1	1	1	2	1	1	1	8
Ma (HPFS) 2019 [39]	0	1	1	1	2	1	1	1	8
Yang (NHS) 2021 [40]	0	1	1	1	2	1	1	1	8
Yang (HPFS) 2021 [40]	0	1	1	1	2	1	1	1	8
Zhang (SWHS) 2013 [19]	0	1	1	1	2	1	1	1	8
Zhang (SMHS) 2014	0	1	1	1	2	1	1	1	8
Zhong 2020	1	1	1	1	2	1	1	0	8

### Association between dietary patterns and HCC risk

#### 1. A priori dietary patterns

##### HEI & AHEI

In our study, we explore two a priori dietary indicators guided by specific guidelines: the Healthy Eating Index (HEI) and the Alternate Healthy Eating Index (AHEI). The HEI is designed to assess adherence to the Dietary Guidelines for Americans (DGAs), which are updated every five years [41]. The AHEI, on the other hand, was initially developed to study the impact of food and nutrients on chronic disease risk and is considered an alternative to the HEI [42]. The primary distinctions between these indices lie in their categorization of alcohol, nuts, and/or legumes, differentiation between white and red and/or processed meats, and consideration of long-term multivitamin use.

**Fig. 2:**
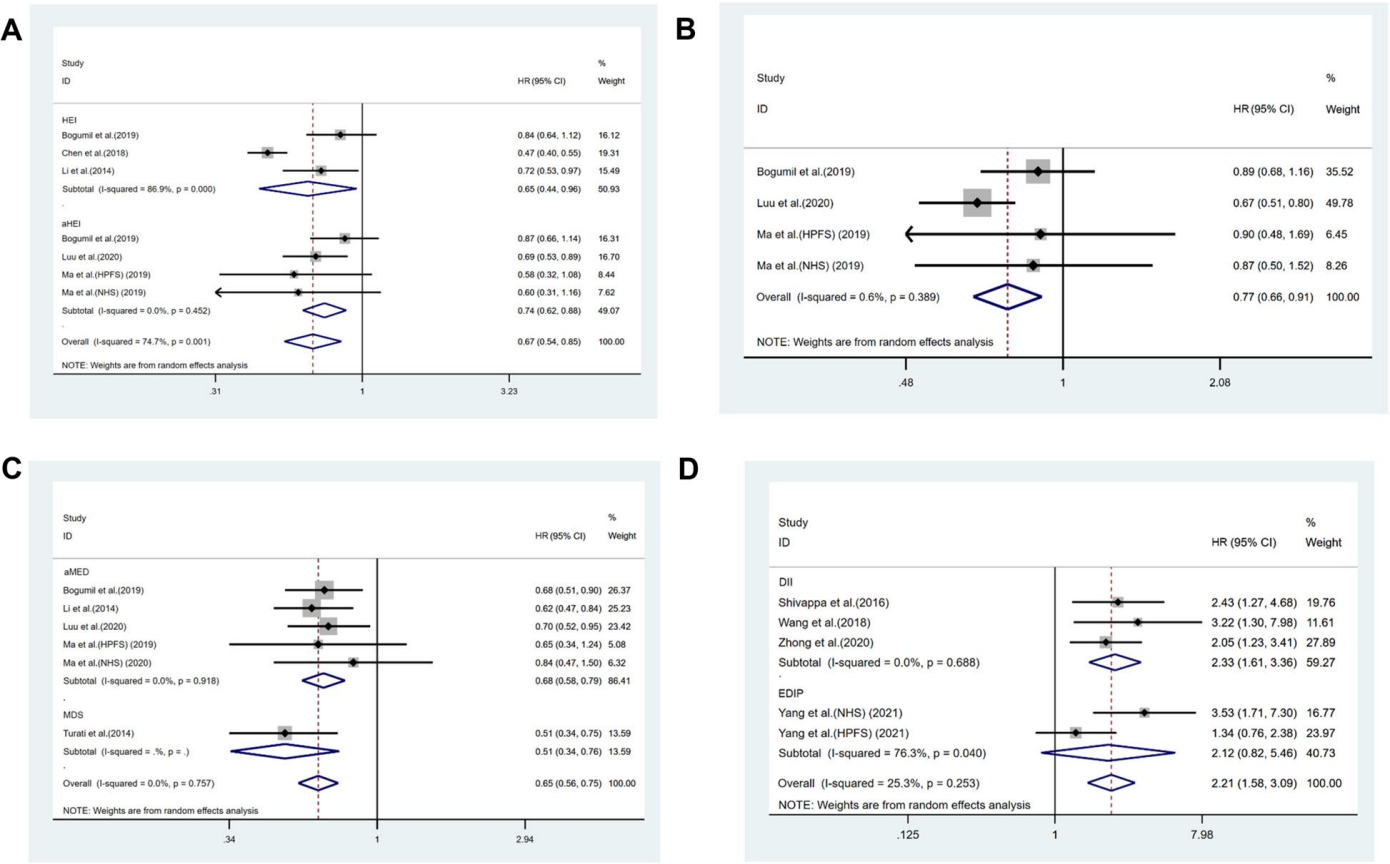
Forest plot depicting the risk of HCC associated with a priori dietary patterns, comparing highest and lowest intake categories. It was stratified by dietary pattern score: **A** HEI & aHEI, **B** DASH, **C** MD, and **D** pro-inflammatory diet. Hazard ratios (HRs) with 95% confidence intervals (CIs) were used to represent associations. The weighted summary effects were derived using a random effects model. Here, squares indicate effect sizes, lines extend to show 95% CIs, and the diamond marks the aggregated effect size. The *p*-value tests for homogeneity in effect sizes across studies, while *I* ^2^ quantifies the proportion of total variation due to heterogeneity. Abbreviations: HEI, Healthy Eating Index; aHEI, Alternative Healthy Eating Index; DASH, Dietary Approaches to Stop Hypertension; aMED, Alternate Mediterranean Diet; MDS, Mediterranean Diet Score; DII, Dietary Inflammatory Index; EDIP, Empirical Dietary Inflammatory Pattern

In Bogumil's study [15], both the HEI and AHEI scores were investigated. To prevent overlap in the sample populations, we conducted a subgroup analysis of these indices. The two dietary scores range as follows: HEI, 0 (lowest adherence) to 100 (highest adherence); AHEI, 0 (lowest adherence) to 110 (highest adherence). Compared to the lowest adherence of the HEI & AHEI dietary patterns, the highest adherence demonstrated a significant reduction in risk, with a pooled Hazard Ratio (HR) of 0.67 (95% CI: 0.54–0.85, *p* = 0.001; see Fig. 2A), and exhibited considerable heterogeneity (*I* ^*2*^ = 74.7%, *p* = 0.001). In the subgroup analysis, a negative correlation was found between the AHEI and HCC risk (HR = 0.74, 95% CI: 0.62–0.88, *p* = 0.001), with no significant heterogeneity (*I* ^*2*^ = 0.0%, *p* = 0.452). This negative correlation was also present in the HEI (HR = 0.65, 95% CI: 0.44–0.96, *p* = 0.029), but with high heterogeneity (*I* ^*2*^ = 86.9%, *p* < 0.001). Sensitivity analysis revealed that this heterogeneity primarily originated from the study by CHEN et al. [16], which was a case–control study and may have been subject to recall bias, unlike the other cohort studies. Upon exclusion of this study, the heterogeneity significantly decreased (*I* ^*2*^ = 0.0%, *p* = 0.648), and the inverse relationship remained significant (HR = 0.76, 95% CI: 0.66–0.86, *p* < 0.001).

##### DASH diet

The Dietary Approaches to Stop Hypertension (DASH) diet, initially developed for the management of hypertension, predominantly consists of a rich variety of fruits, vegetables, and low-fat dairy products. It has been proven to effectively lower blood pressure and modulate levels of low-density lipoprotein (LDL) and high-density lipoprotein (HDL) [43]. In examining the relationship between the DASH diet and the risk of HCC, we observed that the pooled hazard ratio (HR) between the highest and lowest categories of the DASH diet was 0.77 (95% Confidence Interval [CI] 0.66–0.91, *p* = 0.002), indicating a statistically significant association. Moreover, the heterogeneity among different studies was remarkably low (*I* ^*2*^ = 0.6%, *p* = 0.389) (Fig. 2B). Sensitivity analyses, which involved the sequential removal of individual studies, consistently showed robust results, further affirming the reliability of our findings.

##### Mediterranean diet

The Mediterranean diet, an established dietary pattern rooted in cultural practices, focuses on a high intake of plant-based foods, moderate consumption of fish, olive oil, and alcohol, and restricted intake of red or processed meats and dairy [44]. The Mediterranean Diet Score (MDS), introduced by Trichopoulou et al. in 2003 [45], and its alternative version (aMED) are used to measure adherence to this diet. Compared to the MDS, the aMED separates fruits and nuts, removes dairy, and includes only whole grains (instead of all grains) as well as red and processed meats (instead of all meats) [14].

In our analysis, which included six studies (five using aMED and one using MDS), the Mediterranean diet was associated with a significantly reduced HCC risk, with a pooled hazard ratio (HR) of 0.65 (95% CI: 0.56–0.75, *p* < 0.001) (Fig. 2C). This finding was consistent across studies, showing low heterogeneity (*I* ^*2*^ = 0.0%). Specifically, the pooled HR using the aMED score was 0.68 (95% CI: 0.58–0.79, *p* = 0.001), with similarly low heterogeneity (*I* ^*2*^ = 0.0%, *p* = 0.891). The study utilizing the MDS reported an HR of 0.51 (95% CI: 0.34–0.76, *p* < 0.001); however, due to it being the sole study using this score, the results should be interpreted with caution. Sensitivity analysis affirmed the stability of these results.

##### Pro-inflammatory diet

To evaluate dietary inflammatory potential, researchers have developed indices like the Dietary Inflammatory Index (DII) and the Empirical Dietary Inflammatory Pattern (EDIP). The DII is more commonly used, while the EDIP was specifically developed for a subset of the Nurses' Health Study. The DII is based on the intake of up to 45 different dietary components, most of which are macronutrients and micronutrients, including total energy, carbohydrates, protein, total fat, saturated fatty acids, cholesterol, vitamin B12, iron (pro-inflammatory factor), etc. [46]. EDIP was derived on the basis of 39 predefined food groups using stepwise linear regression to identify a dietary pattern most predictive of 3 inflammatory biomarkers (i.e., IL6, CRP, and TNF-alpha receptor-2) [40].

In examining the relationship between pro-inflammatory diets and HCC risk, five studies were analyzed, of which three utilized the DII score and two employed the EDIP score. Our analysis, conducted using a random-effects model, revealed that pro-inflammatory diets significantly increase the risk of HCC, with a pooled hazard ratio (HR) of 2.21 (95% Confidence Interval [CI]: 1.58–3.09, *p* < 0.001) (Fig. 2D), and overall low heterogeneity (*I* ^*2*^ = 25.3%, *p* = 0.253). Specifically, DII studies showed a pooled HR of 2.33 (95% CI: 1.61–3.36, *p* < 0.001) with minimal heterogeneity (*I* ^*2*^ = 0.0%, *p* = 0.688), whereas EDIP studies indicated an HR of 2.12 (95% CI: 0.82–5.46, *p* = 0.121) with significant heterogeneity (*I* ^*2*^ = 76.3%, *p* = 0.040). This heterogeneity, particularly in EDIP studies, could be partly due to gender disparities in study populations, as the Health Professionals Follow-up Study (HPFS) included only males, while the Nurses’ Health Study (NHS) involved only females. Sensitivity analysis affirmed the consistency of these results across studies. Therefore, we performed subgroup analyses to assess whether these associations between pro-inflammatory diets and HCC risk differed by gender. Moderate heterogeneity existed in both subgroups. Sensitivity analysis revealed that this heterogeneity primarily originated from the study by Yang et al. [40]. Different from others’ findings, Yang’s research showed that associations for inflammatory dietary pattern appeared stronger in women than in men. However, Yang’s study included two nationwide cohorts, the other two were case–control studies with limited sample sizes. It is difficult to assert the role of gender and further research is needed.

##### Other a priori dietary patterns

In investigating the links between other a priori dietary patterns and HCC risk, three studies offer insightful contributions. Chen et al.'s research [16] highlights a substantial link between the higher Chinese Healthy Eating Index (CHEI) and a lower risk of HCC, evidenced by an odds ratio of 0.43 (95% CI: 0.38–0.50), although the specific P-value is not disclosed. Conversely, Luu et al. [18] did not demonstrate a significant association between the Healthy Diet Index (HDI) and hepatocellular carcinoma (HCC) risk, indicated by an odds ratio of 0.85 (95% CI: 0.55–1.09, *p* = 0.04). Moreover, Yang et al. [40] discovered that a higher score on the Empirical Dietary Index for Hyperinsulinemia (EDIH) correlates with an increased HCC risk, with a Hazard Ratio of 1.61 (95% CI: 1.06–2.43, *p* = 0.02). They also found a positive association between the Empirical Dietary Insulin Resistance Index (EDIR) and HCC risk, with a Hazard Ratio of 0.62 (95% CI: 1.08–2.42, *p* = 0.02).

#### 2. A posterior dietary patterns

##### Vegetable-based patterns

**Fig. 3:**
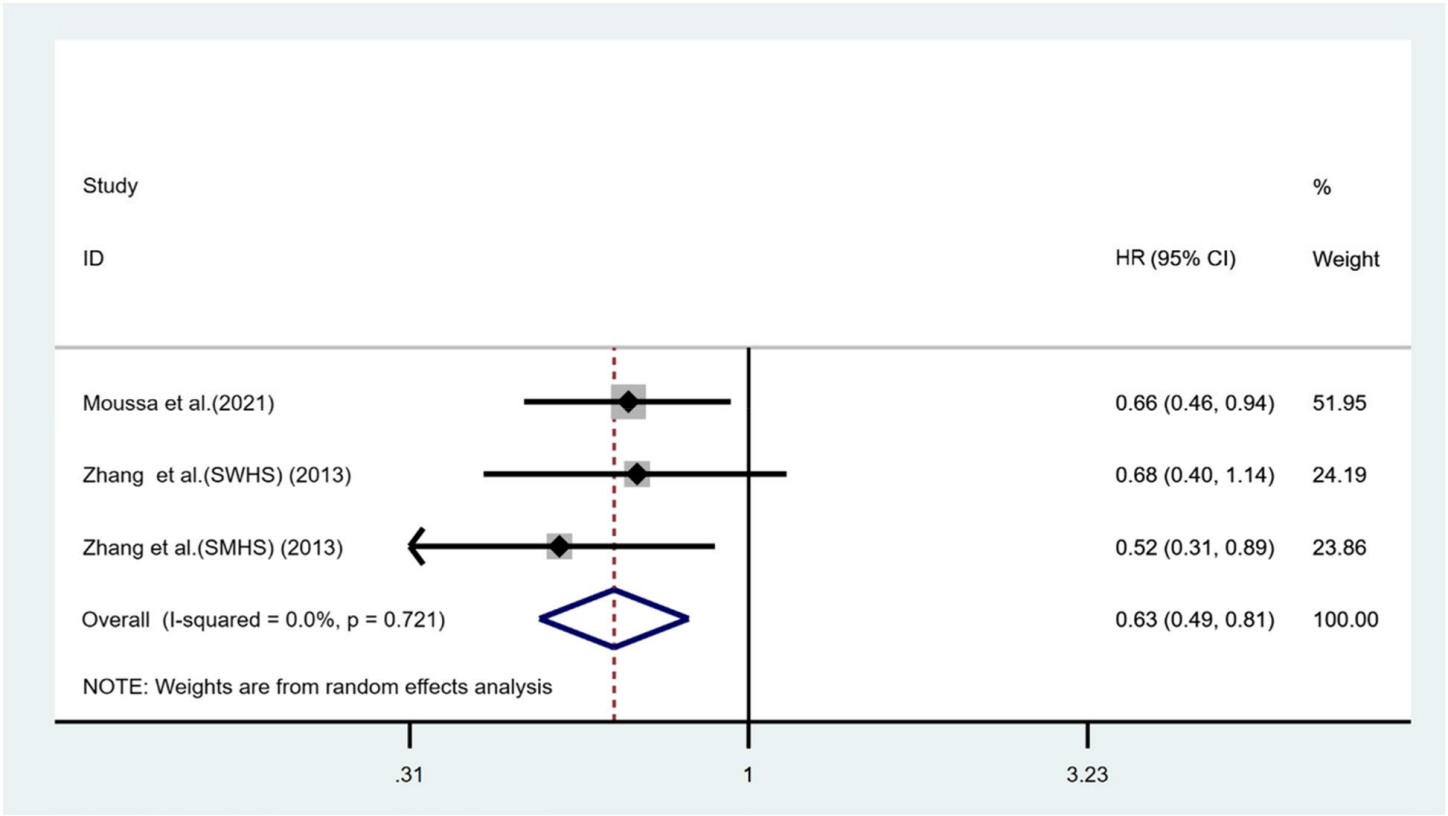
Forest plot illustrating the risk of HCC in relation to vegetable-based dietary patterns, comparing highest and lowest intake categories. Hazard ratios (HRs) with 95% confidence intervals (CIs) were used to represent associations. The weighted summary effects were derived using a random effects model. Here, squares indicate effect sizes, lines extend to show 95% CIs, and the diamond marks the aggregated effect size. The *p*-value tests for homogeneity in effect sizes across studies, while *I* ^*2*^ quantifies the proportion of total variation due to heterogeneity

In examining the association between vegetable-based diets and HCC risk, our analysis comparing the highest and lowest dietary categories revealed a pooled hazard ratio (HR) of 0.63 (95% Confidence Interval [CI]: 0.49–0.81, *p* < 0.001), with minimal heterogeneity observed across the studies (*I* ^*2*^ = 0.0%, *p* = 0.721), as shown in Fig. 3. Further sensitivity analysis, which involved the removal of any individual study, consistently demonstrated the robustness of these results. This finding underscores the potential protective role of a vegetable-based diet in reducing the risk of HCC.

##### Other a posterior dietary pattern

In a study on a posterior dietary patterns, Lan et al. [36] identified three distinct patterns and their associations with HCC risk. The Urban Prudent Dietary Pattern (UPDP), high in dairy, eggs, mushrooms, nuts, soy, and low in refined grains, was associated with a reduced risk of primary HCC (PLC), with an Odds Ratio (OR) of 0.25 (95% CI: 0.18–0.35). The Traditional Cantonese Diet Pattern (TCDP), rich in fruits, vegetables, fish, and herbal teas, also showed a lower PLC risk (OR = 0.61, 95% CI: 0.46–0.82). In contrast, the Meat and Preserved Food Pattern (MPFP) correlated with a higher risk (OR = 1.98, 95% CI: 1.46–2.69). This indicates that UPDP and TCDP are associated with lower PLC risk, while MPFP correlates with higher PLC risk (*p* < 0.01 in all tests).

Moussa's research [17] further indicated a direct link between the Western dietary pattern and HCC risk (OR = 1.79, 95% CI: 1.19–2.69), while Zhang's study [19] found no significant relationship between diets high in fruits or meats and HCC risk. These studies collectively highlight the nuanced relationship between various dietary patterns and HCC risk.

##### Publication bias

The Egger’s tests revealed no statistical evidence of publication bias in our study (Supplementary Materials Fig. 1). However, the number of studies we included was limited, which might result in Egger's test not detecting bias.

## Discussion

Previous independent studies on dietary patterns and HCC risk have shown mixed results for the Mediterranean Diet (MD). A higher score in the Healthy Eating Index (HEI) and Alternate Healthy Eating Index (aHEI), reflecting better adherence to the most authoritative dietary guidelines for the Americans, may reduce HCC risk, with no significant link found between the Dietary Approaches to Stop Hypertension (DASH) diet and HCC [20]. Our meta-analysis indicates that the a priori dietary patterns of aHEI, HEI, DASH, and MD all negatively correlate with HCC risk. Although different approaches are used to obtain optimal scores in HEI, AHEI, MED, aMED, and DASH, all these healthy diets are characterized by a high intake of vegetables, fruits, whole grains, legumes and nuts, as well as a low intake of red meat and processed meat [41, 45, 47]. Foods promoted in these diets are rich in antioxidants and dietary fiber, which can lower HCC risk through multiple mechanisms including antioxidation, improved insulin sensitivity, reduced inflammation, and effects on fat formation and degeneration [48–51]. Conversely, saturated fats and dietary heme iron from red meat may promote HCC by affecting liver lipid distribution and accelerating hepatocyte damage and death. Red and processed meats are also sources of various carcinogens formed during cooking [10, 52, 53].

The DASH diet, while effective for hypertension management, shows a less pronounced effect on HCC risk reduction, possibly due to its emphasis on low-fat dairy products, which can prevent cardiovascular diseases [54]. However, low-fat dairy may increase circulating levels of insulin-like growth factor I (IGF-1) [55, 56], which is conducive to HCC development [57, 58]. Besides, not all versions of the DASH score include a component pertaining to fat consumption [59], whereas fats play a role in HCC progression. Unlike the DASH diet, the MD promotes the intake of monounsaturated fatty acids (MUFA), and adherence to aHEI recommends long-chain (n-3) fats while reducing total polyunsaturated fatty acids (PUFA) intake. MUFAs, primarily from fish, nuts, and olive oil, can reduce liver inflammation, fat formation, oxidative stress, or steatosis [39]. MUFAs, primarily from fish, nuts, and olive oil, can reduce liver inflammation, fat formation, oxidative stress, or steatosis. PUFAs are divided into n-3 (mainly from marine organisms or deep-sea fish) and n-6 (easily obtained from terrestrial animals and plant seeds). n-3 PUFAs have anti-inflammatory effects through various mechanisms. However, the intake of n-3 PUFAs is much lower than that of n-6 PUFAs, whose metabolism can increase pro-inflammatory product levels. These are involved in the progression of non-alcoholic steatohepatitis (NASH) from advanced fibrosis to cirrhosis and eventually HCC, thus total PUFA intake correlates with increased HCC risk [60]. While these diets have been effective in reducing HCC risk, they were initially aimed at managing other chronic diseases, not specifically cancer prevention. Future research should therefore concentrate on formulating dietary patterns explicitly targeted towards cancer prevention.

Evidence from over ten systematic reviews and meta-analyses suggests a significant association between pro-inflammatory diets and a heightened risk of cancer [61–70]. Our study corroborates this, showing that diets high in inflammatory indices notably raise hepatocellular carcinoma risk. These diets are typically rich in saturated fatty acids (SFAs), carbohydrates, and proteins, while being deficient in polyunsaturated fatty acids, flavonoids, and other essential dietary components [37]. Diets high in SFAs are known to induce cellular lipid peroxidation, leading to increased inflammatory responses. This process not only aggravates liver damage but may also play a crucial role in the onset of hepatocellular carcinoma [71]. Furthermore, pro-inflammatory diets are implicated in indirectly boosting the production of tumor-promoting cytokines like IL-6 and TNF. This elevation in cytokine levels results in liver inflammation and activates pathways involving oncogenic transcription factors such as signal transducer and activator of transcription 3 (STAT3), thus amplifying the risk of developing HCC [72].

Three previous studies have examined the link between established dietary patterns and HCC risk [16, 18, 40]. Findings indicate that the Chinese Healthy Eating Index (CHEI) significantly reduces HCC risk, aligning with typical healthy diet components. Conversely, higher scores on the Empirical Dietary Index for Hyperinsulinemia (EDIH) and the Empirical Dietary Insulin Resistance Index (EDIR) are associated with increased HCC risk, suggesting an interaction between diet and the insulin-related metabolic axis [73]. However, no significant correlation was found between the Healthy Diet Index (HDI) and hepatocellular carcinoma (HCC) risk.

Evidence from a posterior research suggests that diets predominantly comprising vegetables may play a role in lowering HCC risk. Contrarily, findings from Zhang's study indicate a lack of significant association between a fruit-centric diet and HCC risk. This observation is consistent with another meta-analysis, which underscores an inverse relationship between increased vegetable consumption and HCC risk, whereas fruit intake does not demonstrate a similar association [74], The potential adverse impact of high fructose content in fruits, linked to liver damage, might negate the otherwise positive effects of fruit consumption. Additionally, research led by Li and colleagues highlights possible detrimental effects of fruit components in HEI-2010 and aMED dietary patterns on HCC risk [38]. These findings underscore the need for cautious interpretation and further exploration in prospective research. Moreover, other studies have identified Western diets, characterized by high intake of red and processed meats, as well as the MPFP dietary pattern, as factors associated with increased HCC risk.

The Ketogenic Diet (KD), characterized by its low-carbohydrate and high-fat regimen, has recently emerged as a dietary pattern of interest. KD functions by limiting carbohydrate intake, which leads to the production of ketone bodies through fatty acid oxidation in vital organs like the liver, heart, gastrointestinal tract, and kidneys. This process turns ketone bodies into the primary energy source for the body. Considering the liver's pivotal role in glucose and lipid metabolism, KD presents a notable potential in HCC prevention and therapy [75]. However, current literature lacks clinical studies that directly correlate the Ketogenic Diet with HCC, underscoring the need for further investigation into KD's specific effects on HCC.

Our meta-analysis, while robust, encounters several limitations. First, despite most studies adjusting for numerous potential confounders that might affect the link between dietary patterns and HCC, the issue of unmeasured and uncontrolled confounding factors in observational studies persists. Importantly, not all studies accounted for every potential confounder, including hepatitis infection. Second, the possibility of recall bias due to differences in dietary recall between cases and controls, along with selection bias in case–control studies' control groups, cannot be completely discounted. Third, there was significant heterogeneity in our findings, as the studies varied in how they divided score ranges when deriving Hazard Ratios (HRs) and Odds Ratios (ORs) based on the highest and lowest quantiles. Fourth, the calculation of diet indices using local food consumption data collected through food frequency questionnaires may not accurately reflect diverse dietary habits across populations. Furthermore, the existence of unpublished studies that do not demonstrate significant associations between dietary patterns and HCC incidence raises the concern of publication bias, especially given our analysis included only English-language publications. Additionally, the constraints of the limited number of studies precluded conducting meta-regression and an assessment for publication bias.

Our meta-analysis reveals that adherence to dietary patterns and indexes such as the Healthy Eating Index (HEI), Alternative Healthy Eating Index (aHEI), Mediterranean Diet (MD), Dietary Approaches to Stop Hypertension (DASH), and vegetable-based diets may lower HCC risk, while inflammatory diets may increase it. However, the global applicability of these findings requires validation through larger-scale cohort studies. Future research should examine these dietary patterns across different populations and cultural contexts and investigate their role in cancer prevention, incorporating both cohort and case–control studies for a comprehensive assessment.

### Supplementary Information

Supplementary Material 1.

## Data Availability

No datasets were generated or analysed during the current study.
