# Supplementary material for: Dietary patterns and hepatocellular carcinoma risk: a systematic review and meta-analysis of cohort and case–control studies
Source: Nutr Metab (Lond). 2024 Jul 11;21:47. doi: 10.1186/s12986-024-00822-y (PMC11241793; doi:10.1186/s12986-024-00822-y)
Supplement: Supplementary file 1 — Supplementary Material 1. [file 12986_2024_822_MOESM1_ESM.pptx]

## Slide 1
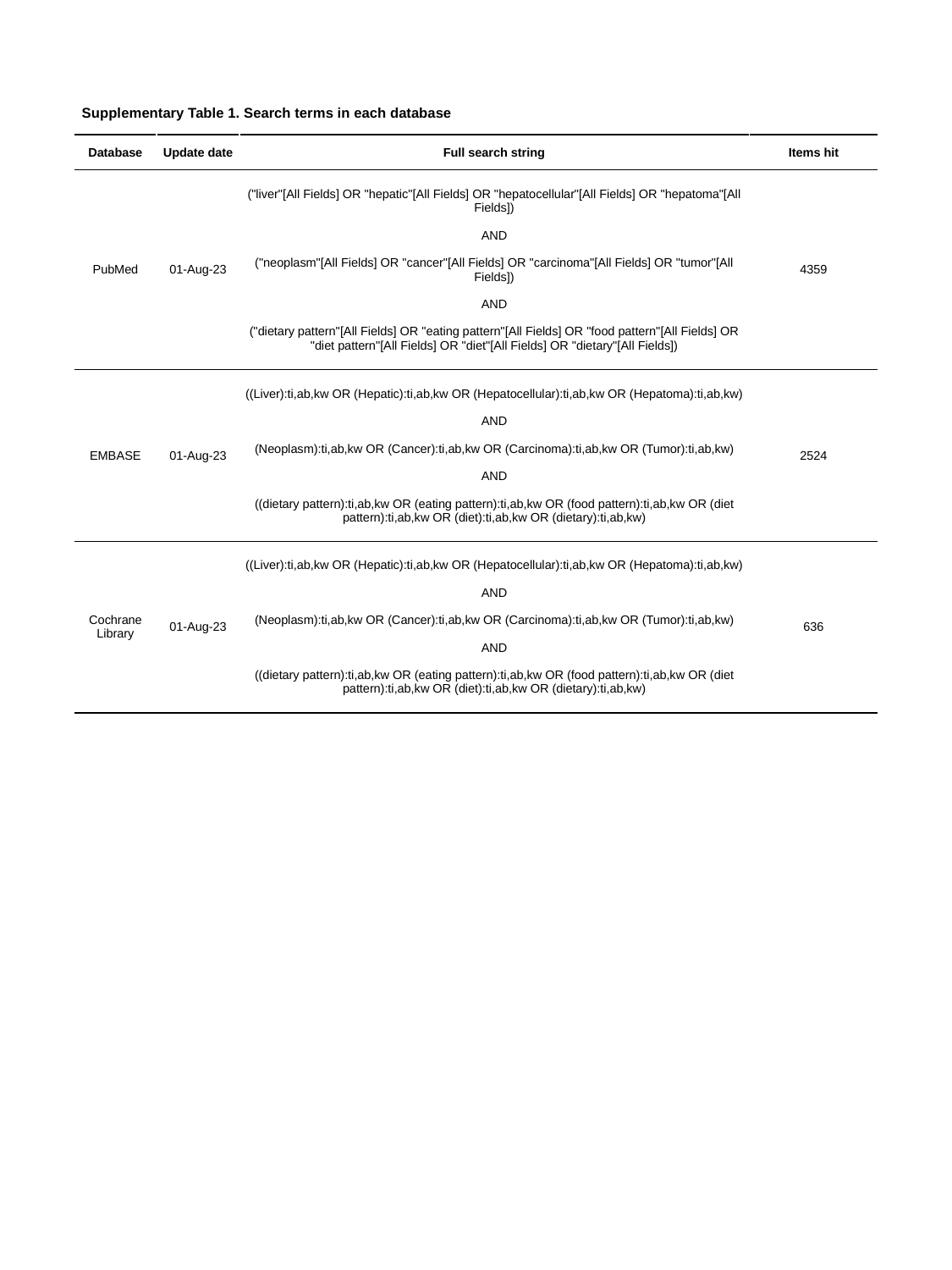

Supplementary Table 1. Search terms in each database
| Database | Update date | Full search string | Items hit |
| --- | --- | --- | --- |
| PubMed | 01-Aug-23 | ("liver"[All Fields] OR "hepatic"[All Fields] OR "hepatocellular"[All Fields] OR "hepatoma"[All Fields]) AND ("neoplasm"[All Fields] OR "cancer"[All Fields] OR "carcinoma"[All Fields] OR "tumor"[All Fields]) AND ("dietary pattern"[All Fields] OR "eating pattern"[All Fields] OR "food pattern"[All Fields] OR "diet pattern"[All Fields] OR "diet"[All Fields] OR "dietary"[All Fields]) | 4359 |
| EMBASE | 01-Aug-23 | ((Liver):ti,ab,kw OR (Hepatic):ti,ab,kw OR (Hepatocellular):ti,ab,kw OR (Hepatoma):ti,ab,kw) AND (Neoplasm):ti,ab,kw OR (Cancer):ti,ab,kw OR (Carcinoma):ti,ab,kw OR (Tumor):ti,ab,kw) AND ((dietary pattern):ti,ab,kw OR (eating pattern):ti,ab,kw OR (food pattern):ti,ab,kw OR (diet pattern):ti,ab,kw OR (diet):ti,ab,kw OR (dietary):ti,ab,kw) | 2524 |
| Cochrane Library | 01-Aug-23 | ((Liver):ti,ab,kw OR (Hepatic):ti,ab,kw OR (Hepatocellular):ti,ab,kw OR (Hepatoma):ti,ab,kw) AND (Neoplasm):ti,ab,kw OR (Cancer):ti,ab,kw OR (Carcinoma):ti,ab,kw OR (Tumor):ti,ab,kw) AND ((dietary pattern):ti,ab,kw OR (eating pattern):ti,ab,kw OR (food pattern):ti,ab,kw OR (diet pattern):ti,ab,kw OR (diet):ti,ab,kw OR (dietary):ti,ab,kw) | 636 |

## Slide 2
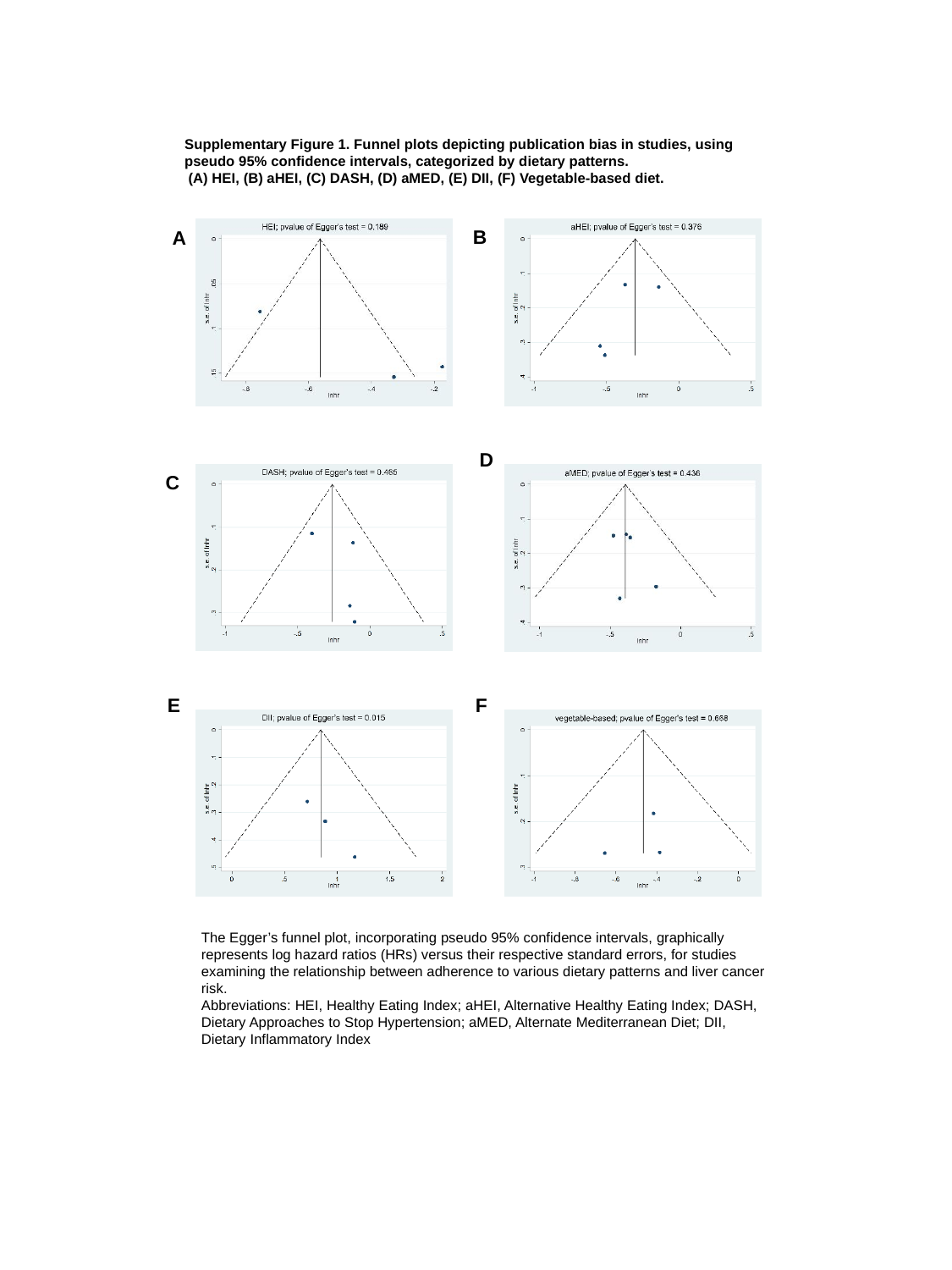

Supplementary Figure 1. Funnel plots depicting publication bias in studies, using pseudo 95% confidence intervals, categorized by dietary patterns.
 (A) HEI, (B) aHEI, (C) DASH, (D) aMED, (E) DII, (F) Vegetable-based diet.
B
A
D
C
E
F
The Egger’s funnel plot, incorporating pseudo 95% confidence intervals, graphically represents log hazard ratios (HRs) versus their respective standard errors, for studies examining the relationship between adherence to various dietary patterns and liver cancer risk.
Abbreviations: HEI, Healthy Eating Index; aHEI, Alternative Healthy Eating Index; DASH, Dietary Approaches to Stop Hypertension; aMED, Alternate Mediterranean Diet; DII, Dietary Inflammatory Index
